# Functional Differences in the Backward Shifts of CA1 and CA3 Place Fields in Novel and Familiar Environments

**DOI:** 10.1371/journal.pone.0036035

**Published:** 2012-04-27

**Authors:** Eric D. Roth, Xintian Yu, Geeta Rao, James J. Knierim

**Affiliations:** 1 Department of Psychology, University of Delaware, Newark, Delaware, United States of America; 2 Department of Neurobiology and Anatomy, University of Texas Medical School at Houston, Houston, Texas, United States of America; 3 Department of Neuroscience and Krieger Mind/Brain Institute, Johns Hopkins University, Baltimore, Maryland, United States of America; Imperial College London, United Kingdom

## Abstract

Insight into the processing dynamics and other neurophysiological properties of different hippocampal subfields is critically important for understanding hippocampal function. In this study, we compared shifts in the center of mass (COM) of CA3 and CA1 place fields in a familiar and completely novel environment. Place fields in CA1 and CA3 were simultaneously recorded as rats ran along a closed loop track in a familiar room followed by a session in a completely novel room. This process was repeated each day over a 4-day period. CA3 place fields shifted backward (opposite to the direction of motion of the rat) only in novel environments. This backward shift gradually diminished across days, as the novel environment became more familiar with repeated exposures. Conversely, CA1 place fields shifted backward across all days in both familiar and novel environments. Prior studies demonstrated that CA1 place fields on average do not exhibit a backward shift during the first exposure to an environment in which the familiar cues are rearranged into a novel configuration, although CA3 place fields showed a strong backward shift. Under the completely novel conditions of the present study, no dissociation was observed between CA3 and CA1 during the first novel session (although a strong dissociation was observed in the familiar sessions and the later novel sessions). In summary, this is the first study to use simultaneous recordings in CA1 and CA3 to compare place field COM shift and other associated properties in truly novel and familiar environments. This study further demonstrates functional differentiation between CA1 and CA3 as the plasticity of CA1 place fields is affected differently by exposure to a completely novel environment in comparison to an altered, familiar environment, whereas the plasticity of CA3 place fields is affected similarly during both types of environmental novelty.

## Introduction

**Figure 1:**
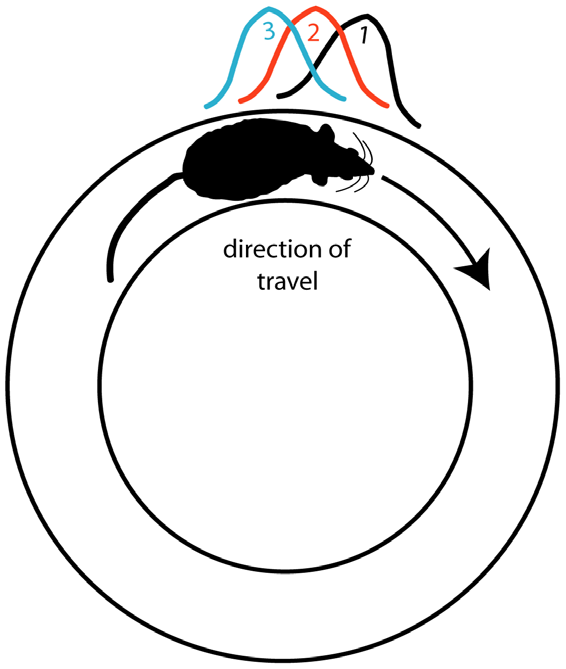
The backward shift of the center-of-mass of a place field. This schematic figure illustrates the lap-by-lap, backward shift of a place field. The rat runs clockwise along a circular track. On lap 1, a place cell fires at a particular location on the track (black). On lap 2, the place field (red) has shifted its center-of-mass slightly backward (relative to the direction of travel of the rat). On lap 3, the field shifts even further backward (green). This phenomenon, originally reported by Mehta and colleagues [7], was shown to depend on NMDA receptors [14] and is thought to be a model of sequence learning [15], [16].

The hippocampus plays an important role in spatial learning and episodic memory [1], [2]. In rats, hippocampal pyramidal cells [3] and granule cells [4] have increased firing rates in distinct spatial locations (i.e., place fields) of the environment. One property associated with plasticity and spatial learning mechanisms is the experience-dependent, backward shift of the center of mass (COM) of the place field (Fig. 1). When rats run in stereotyped routes, the place field COM tends to shift in the direction opposite to the rat's trajectory [5]–[12] (but see [13] for a demonstration of forward shift). This backward shift is NMDA receptor dependent [12], [14] suggesting the involvement of a LTP mechanism. Mehta et al. [7], [9] suggested that the COM shift may reflect the encoding by synaptic weight changes of spatiotemporal sequences of locations of a well-learned route, providing support for predictions of computational models of sequence learning and spatial navigation [15]–[17] and for Hebb's concept of the “phase sequence" [18].

Given the importance of place cells to spatial learning and memory and the potential links between synaptic plasticity mechanisms and experience-dependent place field properties, the dynamic COM shift phenomenon provides an interesting tool for examining functional differentiation within the hippocampus. Computational models, inspired by differences in anatomy, connectivity, and synaptic physiology, suggest unique functional roles for hippocampal subregions [19]–[21]. Experimental studies report differences in ensemble activity between the dentate gyrus and CA regions [22], as well as differences between CA1 and CA3 [5], [23]–[26]. The first major dissociation reported between CA1 and CA3 place fields was a study of the COM-shift phenomenon by Lee et al. [5], who performed simultaneous recordings in CA1 and CA3 in both a stable and a changing environment. In the changing environment, local and distal cues of a standard, familiar environment were rotated in opposite directions (counterclockwise and clockwise, respectively), creating mismatched versions of the standard environment. CA1 and CA3 place fields responded differently to this manipulation, as the COM shift varied between both subregions and environments. In the standard environment, CA1 place fields showed a backward shift whereas CA3 place fields were stable across laps. In the mismatch environments, CA3 place fields showed a backward shift in the first session only (when the mismatch was a novel experience), but were stable in subsequent sessions. Conversely, CA1 place fields (at the population level) did not show a backward shift in the first mismatch session, but showed the effect in subsequent mismatch sessions. Based on their analyses of the related phenomenon of the development of negative skewness in the shape of the place fields [5], [9], Lee et al. [5] concluded that CA3 was specialized for rapid learning and long-term storage of novel spatiotemporal sequences [27] , whereas CA1 was specialized for short-term storage of ongoing sequences for comparison with the long-term memories of sequences stored in CA3 [28].

The mechanisms driving the different patterns of activity in CA3 and CA1 remain unclear. The mismatched cue environment created a variety of remapping phenomena in both regions, as subsets of cells either rotated clockwise, counterclockwise, appeared, disappeared, or developed split-field representations. A follow up study [29] determined that there was no strong relationship between whether a cell remapped and whether it showed a COM shift in the mismatch session. However, the lack of a strong COM shift in the CA1 population in the first mismatch session was shown to be the result of individual cells in CA1 showing both forward and backward shifts, thereby canceling out each other. Lee and Knierim [29] thus suggested that the lack of a coherent response in CA1 may be specific to the cue-conflict situation provided by the mismatch environment. The present study expands on this work to further explore functional differentiation between CA1 and CA3 in the COM-shift phenomenon. We asked whether the differences in COM shifts between CA3 and CA1 reflect a generalized response to a novel environment or whether they are the product of other mechanisms specific to the mismatched environment.

## Materials and Methods

### Ethics Statement

Animal care and surgical procedures were performed according to National Institutes of Health Guidelines and approved by the University of Texas Health Science Center at Houston Institutional Animal Care and Use Committee (protocol # HSC-AWC-04-068).

### Subjects and Surgery

Ten adult male Long-Evans rats were maintained on a 12∶12 light dark cycle at 80–90% of their ad libitum weights, and had ad libitum access to water. For surgical implantation of recording electrodes, rats were anesthetized with an initial dose of 60 mg/kg ketamine and 8 mg/kg xylazine followed by isoflurane inhalation to effect. A microdrive array was centered above the right dorsal hippocampus (3.9 mm posterior to bregma, 3.5 mm lateral to midline). The microdrive array was made up of 14 to 20 tetrodes that were constructed from 4 fine (0.0005 inches) insulated nichrome electrode wires twisted together (Kanthal, Palm Coast). Each electrode was gold-plated to obtain impedances between 200–300 kΩ measured at 1 kHz.

### Training and Environmental Setup

**Figure 2:**
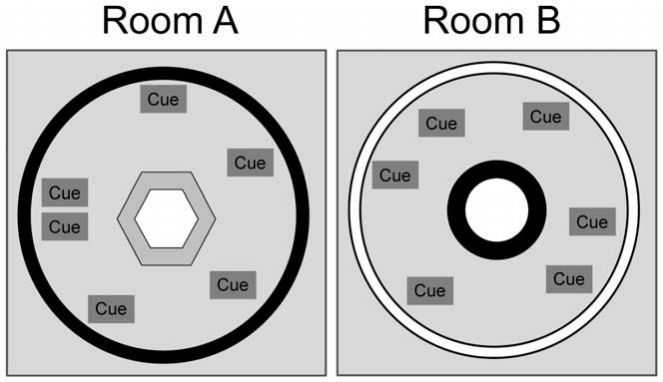
Schematic illustration of the two recording environments. The novel and familiar environments were located in separate rooms. Room A consisted of a black-curtained, circular enclosure with a gray hexagonal track in the center. A variety of cues were placed around the periphery of the enclosure. Room B consisted of a white–curtained, circular enclosure with a black circular track in the center. A completely different set of cues was placed around the periphery in a different configuration.

Two behavioral recording rooms were set up to serve as novel and familiar environments (Fig. 2). Recording areas were cylindrical (2.7 m diameter) with the outer perimeter defined by curtains extending from floor to ceiling. A circular or hexagonal track was placed on a platform in the center of the room. Light was provided by a single 25 W bulb mounted in the center of the ceiling. A commutator with recording tethers and a video camera were mounted on the ceiling slightly offset from the central light. Recording room A was set up with a grey, hexagonal track (each hexagon side = 39.8 cm, track width = 10 cm) with black curtains and a variety of distal cues defining the circular perimeter of the room. The cues were a grey rectangular poster board at 20° (relative to an arbitrarily defined 0°), a triangular cardboard at 80°, a white box positioned on the floor at 80°, a circular white poster board at 160°, a cardboard in the shape of an L at 200°, a white wooden box positioned on floor at 300°, and a white rectangular poster board at 340°. Recording room B was set up with a black, circular track (76 cm outer diameter, track width = 10 cm) with white curtains and a completely different set of distal cues defining the circular perimeter of the room. These cues were a coat rack positioned on the floor at 60°, a square black and white poster board at 100°, a donut-shaped cardboard at 180°, a box positioned on the floor at 230°, and a square black and white poster board picture frame at 340°. The rooms were counterbalanced such that 5 rats were randomly assigned to experience room A as the familiar environment and room B as the novel environment, and the other 5 rats experienced room B as the familiar environment and room A as the novel environment.

After surgery, rats were permitted one week to recover before behavioral training commenced. In the familiar environment, rats were trained to run clockwise on a track to forage for chocolate sprinkles placed at arbitrary locations by an experimenter. Generally 8–10 training sessions of 20 min across ∼10 days were required before the experiment to assure rats met behavioral criteria (i.e., continually foraging for sprinkles with limited interruptions, completing at least 15 laps within 8 min). During this training period, tetrodes were gradually advanced to pyramidal cell layers in CA1 and CA3.

### Experimental Testing

For each rat the experiment consisted of 4 days of testing. Each day the experimental sessions were repeated using the same protocols and environmental conditions. First, rats were permitted to sleep or rest quietly in a dish as hippocampal single-unit recordings were collected for ∼15 min. After the sleep session, rats were transported in the open (without disorientation) into the adjacent behavioral room assigned as the familiar environment (i.e. the environment in which the rat received previous training). Rats were placed on the track at an arbitrary location and 15 clockwise laps were recorded as rats foraged for chocolate sprinkles. Rats were then allowed to rest back in the sleep session room for ∼5 min before they were transported to another adjacent room assigned as the novel environment. On day 1 of the experiment, this was the first time the rats were exposed to this environment. After a 15-lap session in the novel environment, rats were permitted another 5-min rest period before repeating a final, 15-lap session in the familiar environment. After the final session, another baseline sleep session was recorded. These sleep data (before and after the behavioral sessions) were used to assess recording stability.

### Histology

After the experiments ended, small lesions were generated on a subset of tetrodes (10 µA for 15 sec) approximately 24 h before transcardial perfusion with 4% formalin. To ensure that the recording tracks were easily detectable, the brains were partially exposed and allowed to sit in formalin for 4 or more hours with the tetrodes in place, after which the tetrodes were withdrawn, the brains were removed, and they were placed in a 30% sucrose formalin solution. Frozen brains were sectioned at 40 µm on a microtome, mounted, and stained with cresyl violet. Recording locations were assigned by identifying the tetrode tracks across sections and matching them against the known configuration of tetrodes in the recording array.

### Data Analyses

Custom software was used for offline single-unit isolation by examining relative signal amplitudes and other waveform parameters across the four wires of a tetrode. Unit isolation was subjectively categorized on a scale of 1 (very good) to 4 (marginal), based on the overlap of points in the multidimensional waveform parameter space. The unit isolation classification was performed completely independent of the firing properties of the cells. Cells that were judged as marginally isolated were excluded from the quantitative analyses reported here.

To measure the spatial firing characteristics of the cells, the closed-loop tracks were linearized to create 360 equally sized bins (0.66 cm/bin). Firing rate was calculated by dividing the number of spikes fired in each bin divided by the amount of time the rat spent in each bin. Similar to Mehta et al. [7] and Lee et al. [5], place field boundaries were defined by the bins in which the mean firing rate fell below 10% of the peak firing rate of the place field for 20 contiguous bins. A place cell was defined as any cell with a statistically significant (p≤0.01) information score [30] of ≥0.5 bits/spike with ≥50 spikes recorded in the session. Place field width or linear size was defined by the number of bins between field boundaries. Skewness was calculated as the ratio of the third moment of the place field firing rate distribution divided by the cube of the standard deviation [5], [9]. The average track position of the place field was defined as the center of mass of the firing rate distribution within the field boundaries [5], [7], [9]. Calculations of a place field's COM, size, or skewness on a single lap were limited to laps that contained at least 4 spikes within the field boundaries. Simple linear regression analyses were used to investigate lap-by-lap firing patterns. Analysis of variance was used to examine differences between CA3 and CA1 in session-based place field skewness and size. The data generally met the assumptions of these statistical tests, except the session-based field size comparisons marginally violated normality (*P* = 0.035, Kolmogorov-Smirnov). Although the ANOVA is relatively robust to mild deviations in normality and the differences between groups were quite large, we also confirmed these results with nonparametric comparisons (Mann-Whitney). For simplicity we report only the ANOVA results.

### Double rotation experiment

After recordings from the novel environment study were completed, 2 rats from this study were then subjected to double rotation protocols for comparison with the results reported by Lee et al. [5]. Prior to experimental sessions, rats received 3 training sessions (10 minutes per session) for 3 days. Training sessions were similar to the behavioral training for the prior experiment as rats simply performed the same behavioral task (foraging for chocolate sprinkles moving clockwise around the track) within the standard (familiar) environment of the double rotation study. After 3 days of training (i.e. familiarization to the standard environment), 4 days of recording commenced in which 3 sessions (15 laps per session) in the standard environment were interleaved with 2 sessions in a cue-mismatch session. To create the mismatch environment, local cues on the track and distal cues on the wall or on the floor of the room were rotated in opposite directions (clockwise or counterclockwise) creating mismatched versions of the standard environment. Simple linear regressions were used to examine lap by lap changes in COM in CA1 and CA3. Because of the limited sampling of cells in this experiment, the double rotation results are reported only anecdotally.

## Results

Place fields were recorded simultaneously from distal CA3 (primarily CA3a and CA3b (see Fig. 1 in [31] for CA1 and CA3 subdivision references) and proximal CA1 (primarily CA1b and CA1c) as 10 rats ran laps for food reward in one of two rooms (familiar and novel). The data analyses were limited to the novel session and the first familiar session of each day to limit possible confounding interactions between repeated daily exposures to the familiar environment. The number of place cells, combined from all animals for a given behavioral session, that achieved statistically significant spatial criteria and were included in the analyses averaged 32.9±2.7 (SE) cells in CA1 and 28.1±2.8 (SE) in CA3.

### COM Shift Analyses

**Figure 3:**
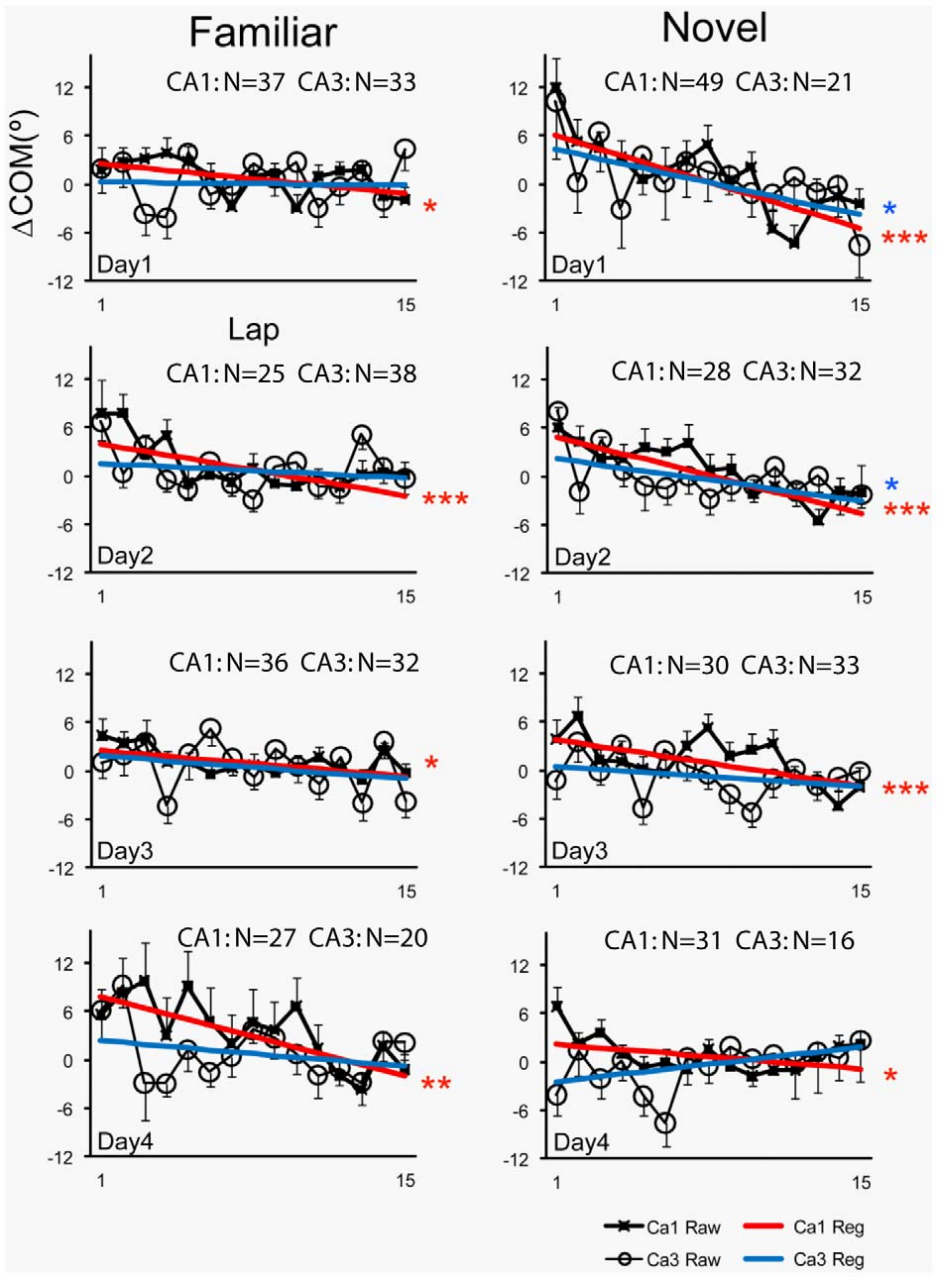
Average lap-based ΔCOM of the CA1 and CA3 place fields in familiar and novel sessions across 4 days. Regression lines are depicted on top of raw data. Rats ran 15 laps in a familiar (left) and novel (right) environment. For each lap, the center of mass (COM) of a place field on that lap was subtracted from the COM of the place field averaged over all laps to produce the ΔCOM measure. Mean and SE are plotted for all cells that met inclusion criteria for a given lap (see Methods). CA3 showed a significant backward shift of the COM only on Days 1 and 2 in the novel environment, whereas CA1 showed a significant backward shift on all days in both environments. Significance levels of linear regression are indicated: *p<0.05, **p<0.005, ***p<0.0005, as well as the number of cells (N) recorded in each session.

To examine the COM shift, we subtracted a cell's place-field COM measured on each lap from the COM of the place field averaged over all laps to generate a ΔCOM measure for each lap [5], [7]. Figure 3 plots the mean (± SE) lap-by-lap ΔCOM for all cells in familiar and novel sessions. Inspection of the raw plots for CA1 (denoted by x marks) and CA3 (denoted by open circles) reveals a large amount of lap-by-lap variability in. ΔCOM, as shown in previous studies [5]–[7]. However, negatively sloped trends in the data were apparent in a number of graphs. In some cases, these trends appear fairly linear (e.g., Day 1 Novel CA1), whereas in other cases the largest shifts appear in early laps and then the graphs flatten or become highly variable in later laps (e.g., Day 4 Familiar CA3). Because there was no *a priori* reason to predict whether the trends would be linear or nonlinear for a particular experimental condition or cell type, we followed our procedures from previous studies [6] and analyzed the data using linear regression in order to quantify general trends and differences between CA1 and CA3. Linear regressions revealed significant (*p*<0.05) backward shifts in CA1 in all familiar and novel sessions (red lines). In contrast, for CA3 cells, we only observed statistically significant backward shifts in the novel sessions on days 1 and 2. Although regressions generally exhibited a negative slope, backward shifting trends of CA3 place fields across the entire session failed to achieve statistical significance in any of the familiar sessions. Thus, similar to Lee et al. [5], the backward shift in CA3 greatly diminished across days as the novel environment became more familiar (days 3 and 4), and was also absent or reduced in the familiar environment. However, Lee et al. [5] reported that CA3, but not CA1, showed the backward shift on day 1 when the novel cue-mismatch condition was experienced for the first time. In contrast, we did not see differences between CA1 and CA3 in the rats' first exposure to a completely novel environment. Thus, the most notable difference between the mismatch environment and a completely novel environment is the presence of a COM shift at the population level in CA1 on day 1 in the novel environment but not in the mismatch environment. The backward shift in CA1 was evident on both circular and hexagonal tracks in the completely novel environment (data not shown).

**Figure 4:**
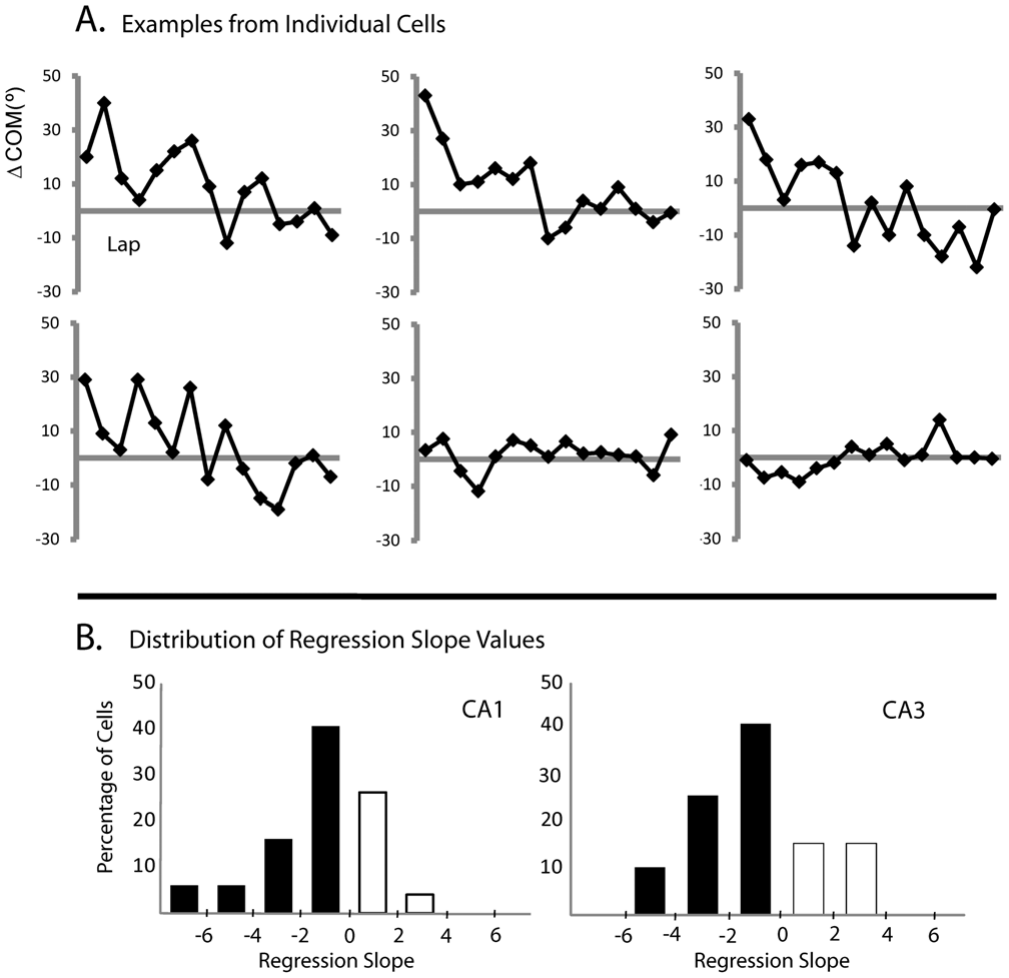
Backward COM shift analyses of individual CA1 cells on day 1 in the novel environment. (A) Examples of lap-by-lap ΔCOM of individual CA1 cells. Most cells showed a backward shift, but a minority showed either no shift or a slight forward shift. (B) Histogram of regression slope values for individual CA1 (left) and CA3 (right) cells. Negative slope indicates a backward-shifting place field, whereas positive slope indicates a forward-shifting place field. In contrast to Lee and Knierim [29], there were fewer cells in CA1 that shifted forward substantially compared to those that shifted backward.

To provide further comparisons between the present study and the study of Lee et al. [5], additional regression analyses of the COM shifts of individual CA1 cells (Fig. 4) in the novel environment revealed that on day 1 most cells (70%) exhibited a backward shift (mean slope = −2.1±0.37 SE), while the remaining 30% of cells had a generally mild positive regression slope (mean = 0.87±0.20 SE). This distribution of slopes was similar in CA3 (Mann-Whitney U: *p* = 0.813). These proportions contrast with the proportions seen in CA1 in the cue-mismatch experiment, in which the numbers of forward- and backward-shifting cells were more similar (∼40/60) and a small number of forward shifting cells had very high, positive slopes [29]. The differences between the studies in the sign and magnitude of the slopes of individual cells thus appears to result in the overall COM shift observed in the novel environment of the present study and the lack of the overall COM shift in the Lee et al. [5] mismatch environment, as the forward- and backward-shifting cells of that study tended to cancel out each other (see below).

### Remapping

**Figure 5:**
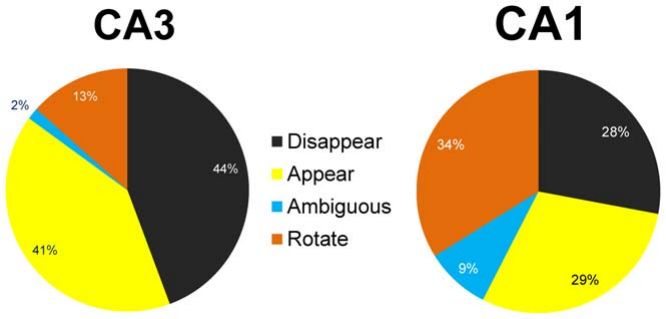
Place field remapping. When transitioning from the familiar to the novel environment, place fields exhibited a variety of remapping behaviors. Some cells maintained a place field in both familiar and novel environments located at different degrees of rotation around the rack (Rotate), while other cells had fields that turned on (Appear), turned off (Disappear), or split into multiple fields (Ambiguous). The pie chart depicts the percentage of cells in CA1 and CA3 that exhibited each type of place field remapping behavior. Only 15% of CA3 cells had place fields in both the familiar and novel environments, compared to 43% of CA1 cells.

Leutgeb et al. [24] reported that CA3 showed a greater degree of complete, “global" remapping between two different rooms compared to CA1. To determine whether our data were consistent with this finding, place fields were classified as rotating, appearing, disappearing, or ambiguous. Rotating cells were defined as any cell that maintained a place field in both novel and familiar environments. Place fields rotated around the track, but the environments were completely different so we have no reference point to assign a degree of rotation. It is thus possible that some of these rotating cells really reflected remapping. Appearing cells did not have a place field in the familiar environment but then developed fields in the novel environment. If cells exhibited a place field in the familiar environment and then lost the field in the novel environment, they were categorized as disappearing. Cells that exhibited split place fields or multiple fields within a single environment were categorized as ambiguous. Remapping results were similar across days (CA1: df = 3, χ^2^ = 7.48, *P*>0.05, CA3: df = 3, χ^2^ = 5.99, *P*>0.05). Daily results were combined to examine overall remapping distributions in CA1 and CA3. Chi-square tests for independence reveal significant differences (df = 3, χ^2^ = 36.8, *P*<0.005) between remapping distributions in CA1 and CA3 (Fig. 5). Compared to CA1 (34% rotate, 9% ambiguous) very few CA3 cells maintained a place field in both familiar and novel environments (13% rotate, 2% ambiguous). Instead, CA3 cells generally responded to the novel environment by losing place fields (44% disappearing) or generating new fields (41% appearing). Thus, similar to Leutgeb et al. [24], even though both CA1 and CA3 showed a high degree of remapping, a greater proportion of CA3 cells had place fields in only one of the two environments compared to CA1.

### Skewness and Size Analyses

**Figure 6:**
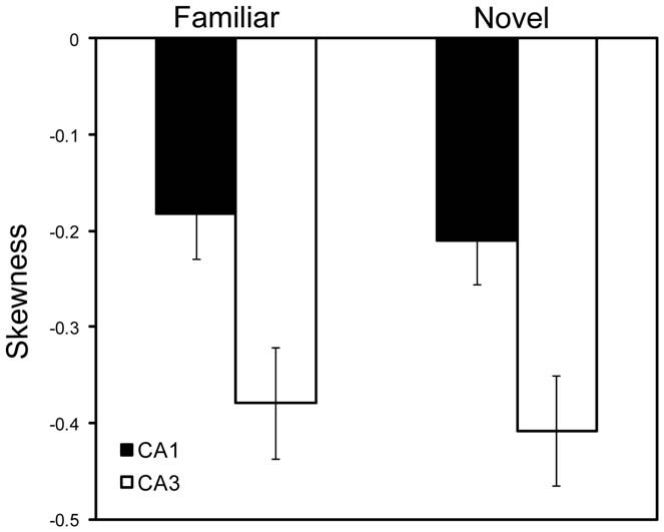
Mean skewness values ± SE for place cells in CA1 and CA3 recorded in the familiar or novel environment averaged across 4 days. CA3 place fields were more negatively skewed than CA1 place fields, but there was no main effect of the environment (familiar vs. novel) and no interaction between the environment and hippocampal subregion.

**Figure 7:**
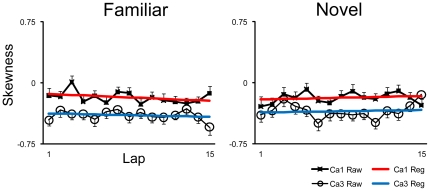
Mean lap-based skewness ± SE of CA1 and CA3 place fields in familiar and novel sessions for all 4 days combined. Regression lines are depicted on top of raw data. No significant regression trends (*P*>0.17) were observed, signifying that the skewness of the place fields did not tend to change over laps in this experiment. When broken down by day, there were no consistent patterns in either hippocampal subregion in either environment (data not shown).

The original reports by Mehta and colleagues [7], [9] reported that increases in the size and shape (i.e., negative skewness) of place fields accompanied the changes in COM. Lee et al. [5] and Yu et al. [6] did not fully replicate these size and skewness changes, and Yu et al. [32] suggested that the COM-shift was a more robust and reliable indicator than size or skewness of the plasticity mechanisms thought to underlie all 3 types of place-field changes. We nonetheless calculated size and skewness changes to see if these effects were present in the current data. We first asked whether the lap-averaged place fields of CA1 and CA3 were negatively skewed. An ANOVA examining session-based skewness values for all days combined, with environment (Familiar or Novel) and subfield (CA1 or CA3) as between-group variables, revealed a highly significant (*P*<0.0001) main effect of subfield, as CA3 cells were more negatively skewed than CA1 cells (similar to [5]) (Fig. 6). No main effects of environment (*P* = 0.594) and no interaction effects (*P* = 0.986) were observed. We next asked whether there were any lap-based changes in the skewness of place fields. There were no consistent patterns across days in familiar or novel sessions (data not shown). CA3 did not show a significant change in skewness over laps in any session (familiar or novel), whereas CA1 showed a significant increase in negative skewness over laps in only day 3 of the familiar environment. Simple linear regression analyses on all days combined for CA1 and CA3 in both familiar and novel environments reveal no linear skewness relationships (*P*>0.17) across laps (Fig. 7).

**Figure 8:**
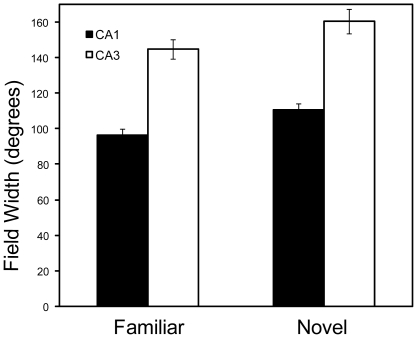
Mean field width values ± SE for place cells in CA1 and CA3 recorded in the familiar or novel environment averaged across 4 days. CA3 place fields were on average larger than CA1 place fields, and in both regions the place fields were slightly larger in the novel compared to the familiar environment. There was no significant interaction between the environment and hippocampal subregion.

**Figure 9:**
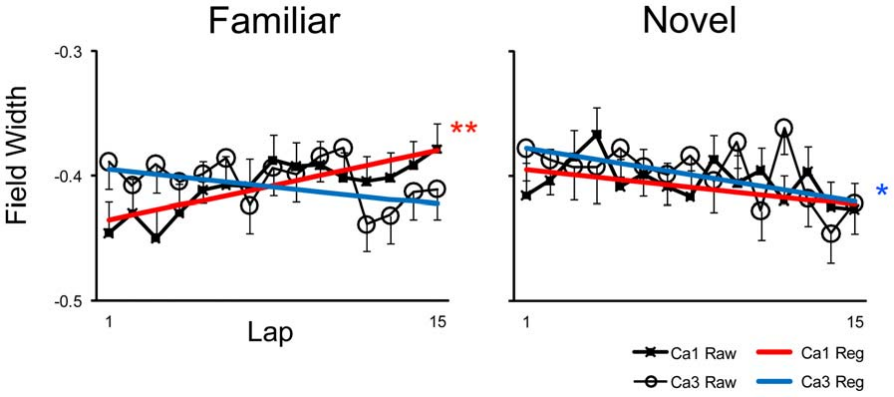
Mean normalized lap-based field width ± SE of CA1 and CA3 place fields in familiar and novel sessions for all 4 days combined. Regression lines are depicted on top of the raw data. CA1 place fields tended to increase in size over laps in the familiar environment, but not in the novel environment. Conversely, CA3 place fields tended to decrease their size, but this effect was not significant in the familiar environment and was only marginally significant in the novel environment. Significance levels of linear regression are indicated: *p = 0.055, **p<0.005.

We performed the same analyses on the place-field size (width) measurements. An ANOVA examining session-based, place-field size values with environment (Familiar or Novel) and subfield (CA1 or CA3) as between group variables revealed highly significant main effects for both subfield (*P*<0.0001) and environment (*P*<0.003). Place fields in CA3 were consistently larger than CA1 fields. Additionally, place fields in novel environments were slightly larger than fields in familiar environments for both subfields (Fig. 8). No interaction effects (*P* = 0.856) were observed. Lap-by-lap field width analyses did reveal some generalized patterns across days. Simple linear regression analyses on all days combined for CA1 and CA3 reveal that CA1 exhibited a significant (*P*<0.005) increase in field width across laps in the familiar environment but not in the novel environment (Fig. 9). Conversely CA3 place fields exhibited a weak trend to become slightly smaller over laps in the novel environment (*P* = 0.055). On individual days, CA1 showed a significant increase in place-field size in the familiar environment only on days 2 and 4, and on none of the days in the novel environment. CA3 place fields exhibited a significant decrease only on day 4 in the novel environment and on day 3 in the familiar environment (data not shown). Thus, unlike the COM shift analysis, the patterns of change in place field size and skewness were not consistent across days and within areas, making interpretation of these parameters difficult.

## Discussion

### COM shift

Lee et al. [5] reported a double dissociation between CA3 and CA1 place fields in the time course of demonstrating the backward COM-shift in a newly reconfigured environment. The present study tested whether this double dissociation generalized to a completely novel environment. Our results replicated most aspects of the Lee et al. [5] findings, but showed a significant difference with that study in the first exposure to the novel environment. In both studies, CA3 place fields did not show a significant backward shift in the familiar environment (as measured by the linear regression analysis). In the novel environment of both studies, CA3 place fields showed a backward shift in the initial sessions, but the shift effect was no longer present in the later sessions (as the novel environment became more familiar). CA1 place fields, in both studies, showed the backward shift in all familiar sessions and in the later novel sessions. However, the two studies differed in terms of the CA1 results on the rats' very first experience with the novel environments. When the novelty consisted of a rearrangement of the familiar cue set (cue mismatch, or double rotation, sessions), CA1 place fields on average did not shift backward [5]. In the present study, however, when the novelty consisted of a completely different room, with an entirely new set of spatial landmarks and behavioral track, CA1 place fields shifted backward over laps just as they did in all other sessions. Thus, the effect of novelty on CA1 did not generalize across the two studies, suggesting that the dissociation between CA3 and CA1 on the first mismatch session [5] had less to do with novelty *per se* than with the precise manipulation employed in that study. (A recent study by Navratilova and colleagues [33] on the development of directional firing of place cells also reports anecdotally a backward shift in the COM of both CA1 and CA3 place fields on the first day of exposure to a novel environment.)

It is conceivable that the difference in results between the double rotation vs novel environment experiments was due to differences in uncontrolled variables, such as how the animals were trained, between the two studies, rather than the novelty manipulation itself. We trained 2 of the rats in the same double-rotation environment after the recordings for the present experiment were concluded. Because the number of subjects and cells were so few, we were unable to perform a rigorous, statistical analysis of these results. However, in general, we replicated the double-rotation results of Lee et al. [5], in that we saw a dissociation between CA1 and CA3 in the COM-shift on Day 1 of the mismatch environment (i.e., CA3 displayed the backward shift and CA1 did not; not shown). Thus, we think it is unlikely that the differences between the double rotation manipulation and the novel environment manipulation were due to uncontrolled variables between the two studies.

In both studies, CA3 cells initially showed a backward shift in the novel environments, but then lost the effect over 1–2 days as the rats gained experienced in the new environment. As argued by Lee et al. [5] and Knierim et al. [28], this pattern of results suggests that the CA3 place fields rapidly encode the new spatial sequences of place fields in a new environment and then maintain these sequence memories in their synaptic weights over repeated exposures to the environment, forming a long-term memory of the sequences. Although there is some indication that CA3 fields on average may shift backward very rapidly (in the first 1–3 laps) in some familiar sessions (Fig. 3), this effect is inconsistent across sessions and it is unclear whether it is a real result or a statistical artifact. If it is real, then the rapid shift backward may reflect a “savings" mechanism that is still consistent with the notion that long-term memory of the sequences is stored in the CA3 network. CA1, in contrast, shows the backward shift robustly in all familiar and novel sessions, which suggests that the CA1 sequence memory is transient [7]. The short-term storage in CA1 may be related to its hypothesized functions as a comparator between entorhinal cortex representations about the current state of the world and stored memories in CA3 [5], [23], [34]–[39].

Lee et al. [5] originally interpreted the difference between CA3 and CA1 on the first mismatch session as an indication that CA3 was specialized for rapid learning, whereas CA1 showed plasticity only after a delay. This interpretation was consistent with the results of Nakazawa et al. [27], who showed that mice with genetic knockout of the NMDA receptor in CA3 showed deficits in immediate learning of a new goal location, but were unimpaired in recalling familiar locations days later. A subsequent analysis by Lee and Knierim [29] showed, however, that individual CA1 cells showed a robust backward shift in the first mismatch session. However, approximately equal numbers of CA1 cells showed a robust forward shift, thereby canceling out the backward shift when the data were averaged over the population. They reinterpreted the results in terms of the hypothesized differences in pattern completion/generalization between the recurrent network of CA3 and the feedforward network of CA1 [19], [40]–[43]. In the cue-mismatch session, they hypothesized that individual place fields were driven by conflicting cue sets. CA1 place fields, lacking a recurrent collateral system and associated attractor dynamics, reacted in different ways, with some fields shifting forward and others shifting backward, resulting in a flat relationship between lap number and place field COM when the fields were averaged at the population level. CA3 place fields, on the other hand, were hypothesized to react coherently to the cue-conflict by shifting backward as an ensemble (similar to the coherent control of the place fields by the set of local cues; [23]). In the present study, there was no conflict between the novel and familiar environments, as each environment had a unique set of spatial landmarks and distinct recording tracks. Correspondingly, most CA1 and CA3 cells remapped the novel environment. We suggest that, under these conditions, there was no conflict imposed on individual cells to rotate clockwise or counterclockwise in the first exposure to the novel environment. Thus, due presumably to the LTP mechanisms, most individual CA3 and CA1 place fields shifted backwards, resulting in the population-based backward shift of place fields in both regions on the first exposure to the novel environment.

### Remapping

Another indicator of functional differentiation between CA1 and CA3 can be observed in the remapping data. In CA1 about 41% of cells maintained active place fields in both the familiar and novel environments. Many of these cells spatially remapped as fields rotated around the track. In contrast, only 15% of CA3 cells maintained place fields in both environments. Rather than rotate around the track, most CA3 fields (85%) either appeared or disappeared when introduced to the novel environment. This is strikingly different to how CA3 fields respond to the mismatched environment [23] in which most fields rotated with the mismatched cues. Another striking difference is the percentage of ambiguous cells that developed split field representations or were otherwise difficult to cleanly characterize as having remapped or rotated their place fields with one set of cues or the other. The percentages in the present study (CA1 = 9%, CA3 = 2%) are less than those from a mismatched environment (CA1 = 36.7%, CA3 = 17.7% [29]. Perhaps the mismatched environment creates conflicts between pattern separation and pattern completion processes often observed in response to graded changes in a given environment [22], [44], [45]. Although mechanistic details are yet unclear, differences in remapping trends further suggest that the hippocampal circuitry responds very differently to the 2 contrasting experimental paradigms.

### Size and Skewness

Using the same place field definition criteria, we obtained very similar results to Lee et al. [5], despite differences in experimental paradigms (mismatch versus novel). In both mismatch and novel environments, field widths were larger than in the familiar or standard environment, and place fields in CA3 were significantly larger than those in CA1. The larger place fields in CA3 may be explained by the location (e.g. proximal versus distal CA3) of the recording electrodes, as proximal CA3 fields are smaller than distal CA3 fields [46]; see [5] for discussion). Similar to Lee et al. [5], CA3 place fields were more negatively skewed than CA1 fields, and no differences were observed in session based skewness results between environments (familiar versus novel). Thus, session based size and skewness results from the mismatched environment seem to generalize to the novel environment in the present study.

Similar to patterns of changes in field size previously reported by Mehta et al. [9], lap based size results revealed an experience dependent increase in field size across laps in CA1 in the familiar environment, which coincided with the experience dependent backward shift. However, in the novel environment, an expansion of CA1 fields was no longer observed despite a continued backward shift in COM. Additionally, significant experience dependent skewness patterns were rarely observed in either subregion (CA1, CA3) or environment. In contrast to CA1, in CA3 we did not observe experience dependent place field expansion in either environment. More specifically, we generally observed a weak trend in both environments for a subtle place field contraction across laps. Although further studies would be required to properly evaluate the statistical and biological relevance of the observed experience dependent place field contraction, these results continue to highlight physiological differences between CA1 and CA3. Overall, our results generally suggest that plasticity mechanisms related to experience dependent field expansion are context- and subregion-dependent, but detailed relationships between field size, skewness, and COM shift remain unclear.

In summary, this is the first study using simultaneous recordings in CA1 and CA3 to compare and contrast place field COM shift, field size, skewness, and remapping results in truly novel and familiar environments. Similar to Lee et al. [5], the present study presents *in vivo* neurophysiological data that further demonstrates functional differentiation between CA1 and CA3. In particular, the paper reaffirms the notion that CA3 place fields show a backward shift in the first sessions of exposure to a novel environment, but the phenomenon diminishes in the CA3 place fields as the environment becomes increasingly familiar. In contrast, CA1 place fields show the backward shift in both familiar and completely novel environments, as the place fields appear to “reset" back to their original locations in between recording days. If the backward shift reflects the storage of sequence information in the synaptic weights between place fields [7], [15], [39], which under certain circumstances is reflected as a negative skewness of place fields, these results are consistent with the proposal by Lee et al. [5] that CA3 is the site of long-term storage of these sequences. CA1, on the other hand, appears to maintain these sequences for less than a day, perhaps allowing it to encode new sequences that can then be compared to the long-term stored sequences in CA3 [28].
